# Supplementary material for: Quantifying PON1 on HDL with nanoparticle-gated electrokinetic membrane sensor for accurate cardiovascular risk assessment
Source: Nat Commun. 2023 Feb 2;14:557. doi: 10.1038/s41467-023-36258-w (PMC9895453; doi:10.1038/s41467-023-36258-w)
Supplement: Supplementary file 1 — Supplementary Information [file 41467_2023_36258_MOESM1_ESM.pdf]

Supplementary Information:

PON1-HDL as a Cardiovascular Disease Marker: Precise Quantification with a Nanoparticle-Gated Electrokinetic Membrane Sensor

Sonu Kumar[1], Nalin Maniya[1], Ceming Wang[1], Satyajyoti Senapati[1*] and Hsueh-Chia Chang[1*]
[1]Department of Chemical and Biomolecular Engineering, University of Notre Dame, Indiana 46556, United States.
*Corresponding author: ssenapat@nd.edu, hchang@nd.edu

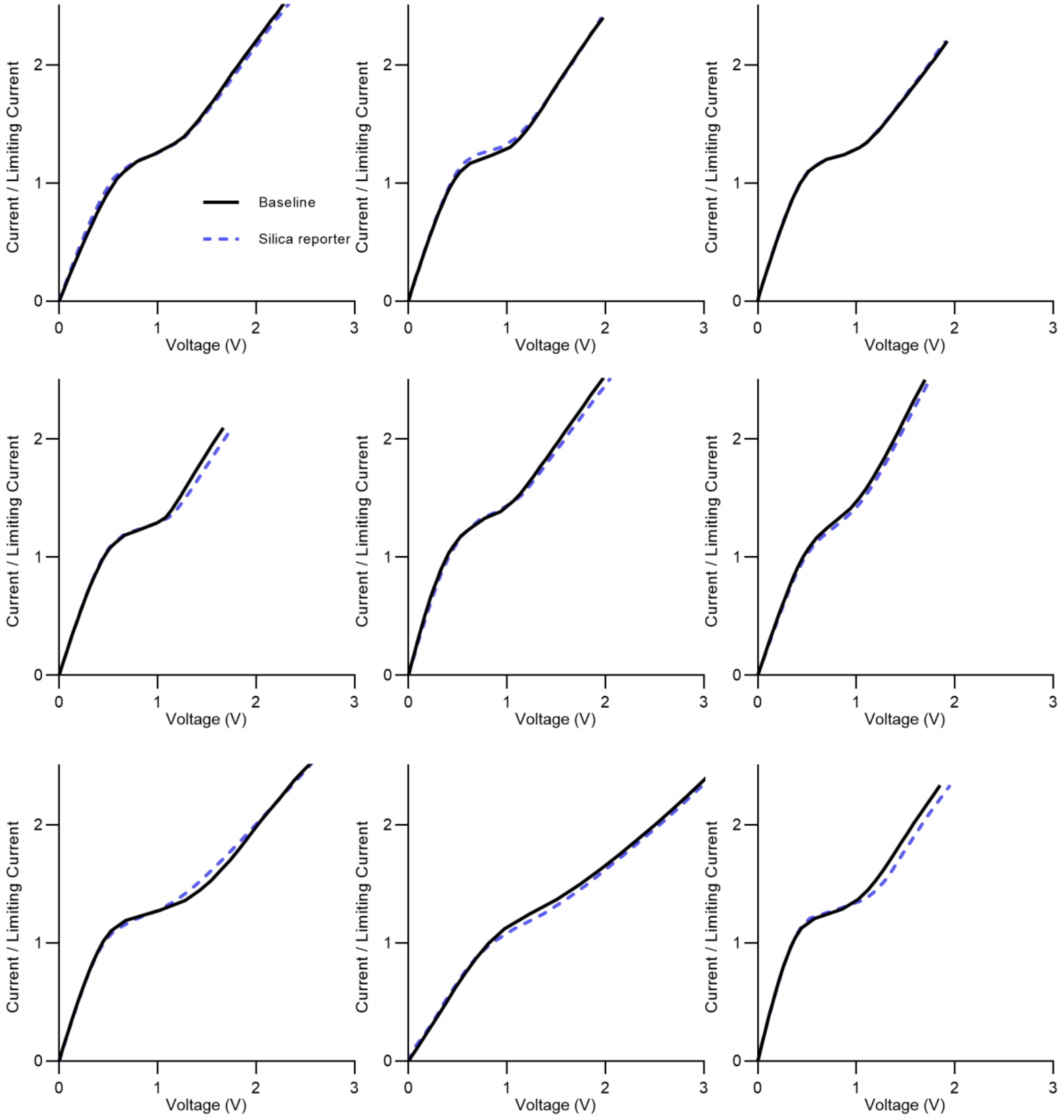

Supplementary Figure 1: **Nine independent cases for Limit of Blank (LOB) calculation using PBS as the blank.** The solid black line represents the baseline while dashed blue line represents the CVC curve after silica nanoparticle incubation and wash as shown in top left legend.

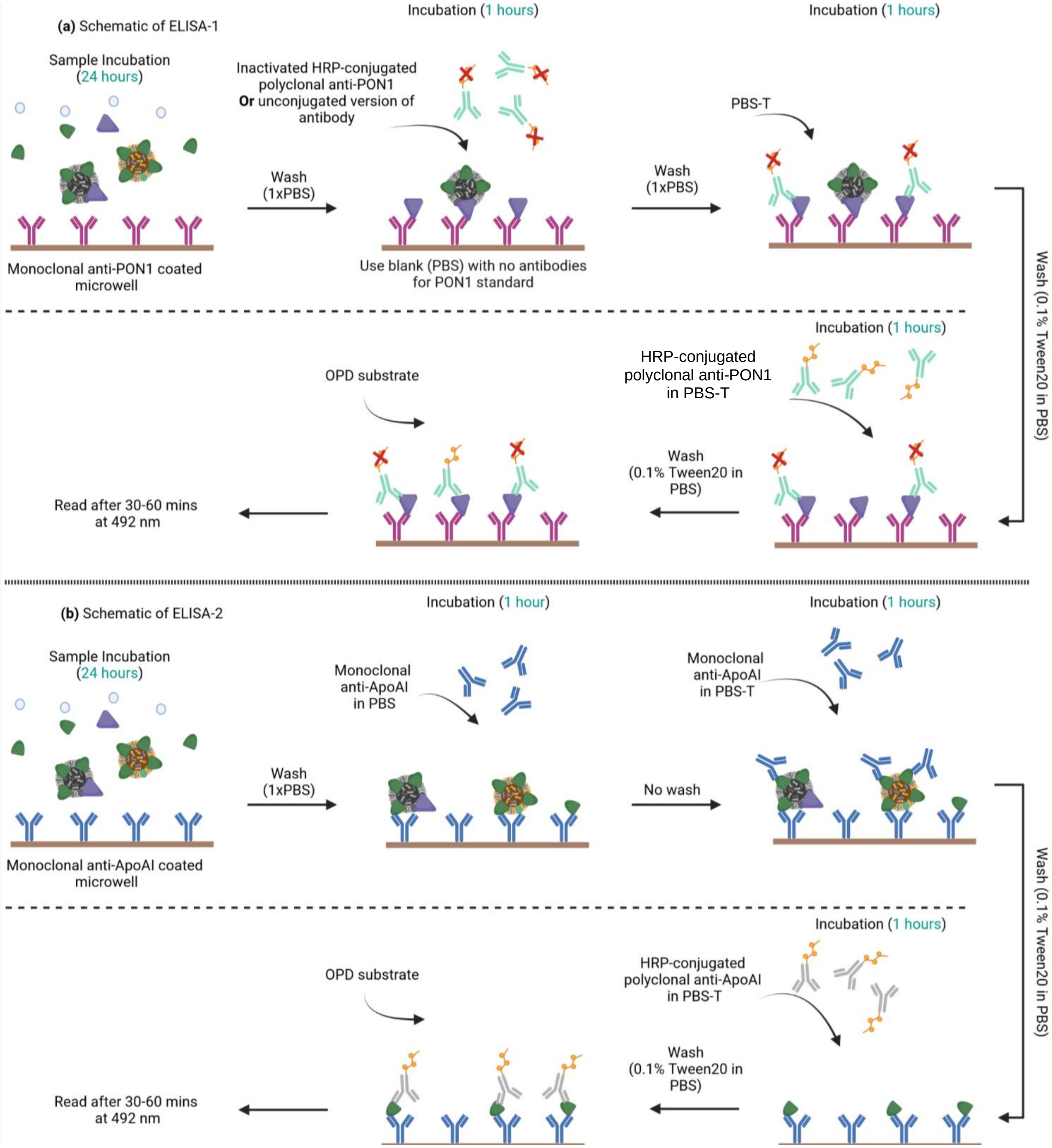

Supplementary Figure 2: **Schematics of our elaborate and novel ELISA schemes** – (a) ELISA-1 and (b) ELISA-2 used to benchmark NGEMS in our study. Schematic created with biorender.com.

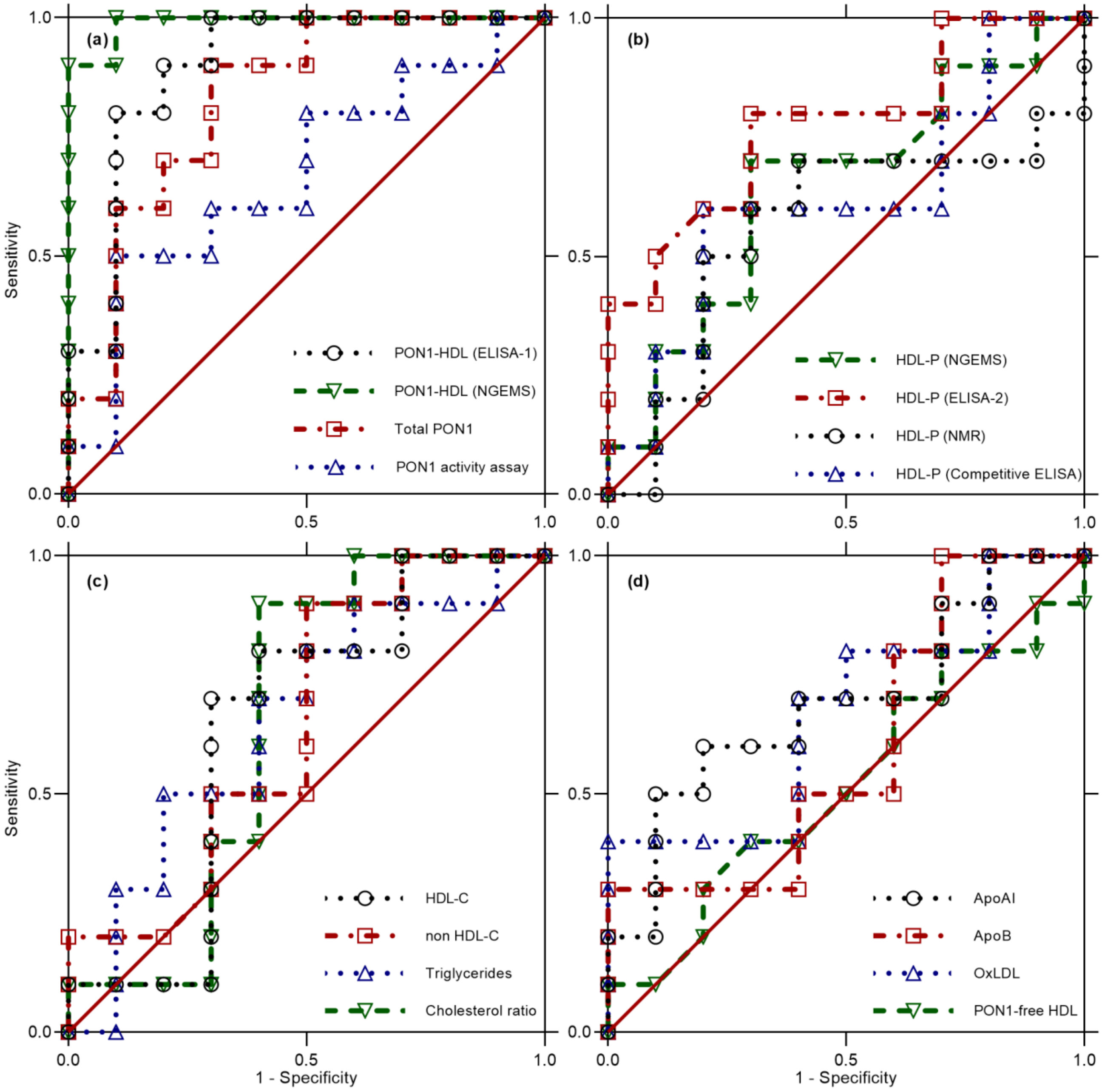

Supplementary Figure 3: **Receiver Operating Characteristic (ROC)** curve for (a) PON1-HDL (NGEMS and ELISA-1), total PON1 and PON1 activity, (b) HDL-P (from NGEMS, ELISA-2, 1H-NMR and Competitive ELISA), (c) HDL-C, non HDL-C, triglycerides and cholesterol ratio, and (d) ApoAI, ApoB and Oxidized LDL from ELISA and PON1-free HDL from NGEMS in distinguishing the coronary artery disease (CAD) group and control group.
