# Supplementary material for: Quantifying PON1 on HDL with nanoparticle-gated electrokinetic membrane sensor for accurate cardiovascular risk assessment
Source: Nat Commun. 2023 Feb 2;14:557. doi: 10.1038/s41467-023-36258-w (PMC9895453; doi:10.1038/s41467-023-36258-w)
Supplement: Supplementary file 3 — Reporting Summary [file 41467_2023_36258_MOESM3_ESM.pdf]

nature portfolio

Corresponding author(s): Prof. Hsueh-Chia Chang
Dr. Satyajyoti Senapati

Last updated by author(s): Dec 28, 2022

Reporting Summary

Nature Portfolio wishes to improve the reproducibility of the work that we publish. This form provides structure for consistency and transparency in reporting. For further information on Nature Portfolio policies, see our Editorial Policies and the Editorial Policy Checklist.

Statistics

For all statistical analyses, confirm that the following items are present in the figure legend, table legend, main text, or Methods section.

n/a	Confirmed	
[ ]	[x]	The exact sample size (*n*) for each experimental group/condition, given as a discrete number and unit of measurement
[ ]	[x]	A statement on whether measurements were taken from distinct samples or whether the same sample was measured repeatedly
[ ]	[x]	The statistical test(s) used AND whether they are one- or two-sided<br>*Only common tests should be described solely by name; describe more complex techniques in the Methods section.*
[x]	[ ]	A description of all covariates tested
[ ]	[x]	A description of any assumptions or corrections, such as tests of normality and adjustment for multiple comparisons
[ ]	[x]	A full description of the statistical parameters including central tendency (e.g. means) or other basic estimates (e.g. regression coefficient) AND variation (e.g. standard deviation) or associated estimates of uncertainty (e.g. confidence intervals)
[ ]	[x]	For null hypothesis testing, the test statistic (e.g. *F*, *t*, *r*) with confidence intervals, effect sizes, degrees of freedom and *P* value noted<br>*Give P values as exact values whenever suitable.*
[x]	[ ]	For Bayesian analysis, information on the choice of priors and Markov chain Monte Carlo settings
[x]	[ ]	For hierarchical and complex designs, identification of the appropriate level for tests and full reporting of outcomes
[x]	[ ]	Estimates of effect sizes (e.g. Cohen's *d*, Pearson's *r*), indicating how they were calculated

Our web collection on statistics for biologists contains articles on many of the points above.

Software and code

Policy information about availability of computer code

Data collection	Gamry Potentiostat Software, Nikon Elements for Confocal Imaging, TopSpin 3.x, COMSOL 6.0
Data analysis	MATLAB R2020b, GraphPad Prism 9, ImajeJ 1.53k

For manuscripts utilizing custom algorithms or software that are central to the research but not yet described in published literature, software must be made available to editors and reviewers. We strongly encourage code deposition in a community repository (e.g. GitHub). See the Nature Portfolio guidelines for submitting code & software for further information.

Data

Policy information about availability of data

All manuscripts must include a data availability statement. This statement should provide the following information, where applicable:

- Accession codes, unique identifiers, or web links for publicly available datasets
- A description of any restrictions on data availability
- For clinical datasets or third party data, please ensure that the statement adheres to our policy

Source data files and uncropped/unprocessed confocal images are provided in the supplementary information.

Human research participants

Policy information about studies involving human research participants and Sex and Gender in Research.

Reporting on sex and gender	The sample size is not large enough to do gender or sex based analysis.
Population characteristics	50% of plasma samples belonged to biological males and other 50% to biological females of Non-hispanic white ethnicity in the 60-70 age group.
Recruitment	Plasma samples were commercially procured through Precision for Medicine.
Ethics oversight	University of Notre Dame

Note that full information on the approval of the study protocol must also be provided in the manuscript.

Field-specific reporting

Please select the one below that is the best fit for your research. If you are not sure, read the appropriate sections before making your selection.

☒ Life sciences ☐ Behavioural & social sciences ☐ Ecological, evolutionary & environmental sciences

For a reference copy of the document with all sections, see nature.com/documents/nr-reporting-summary-flat.pdf

Life sciences study design

All studies must disclose on these points even when the disclosure is negative.

Sample size	The biomarker need to be validated was PON1-HDL and we wanted its AUC 95% CI to be reasonably small. For our current sample size, it was within 0.94-1 which is reasonably good for a pilot study involving PON1-HDL.
Data exclusions	No data was excluded.
Replication	Each experiments were replicated at least three times except cholesterol tests from standard assays that were only duplicated and confocal images that were done once for each case due to acceptable error margin of our platform but repeated for several concentrations.
Randomization	Deidentified human plasma samples were obtained from Precision for Medicine for both the control and coronary artery disease. Each sample was assigned a number 1-20 randomly to blind the experimenter. No randomization was done for the calibration curves, and pure HDL samples of known concentrations were used.
Blinding	Coronary artery disease and healthy samples were obtained from Precision for Medicine. Upon arrival, they were delabeled and randomized by Satyajyoti Senapati and experiments performed by Sonu Kumar. SK performed all experiments for all twenty samples (including the other standard assays) and only then blind was revealed

Reporting for specific materials, systems and methods

We require information from authors about some types of materials, experimental systems and methods used in many studies. Here, indicate whether each material, system or method listed is relevant to your study. If you are not sure if a list item applies to your research, read the appropriate section before selecting a response.

Materials & experimental systems

n/a	Involved in the study	
☐	☒	Antibodies
☒	☐	Eukaryotic cell lines
☒	☐	Palaeontology and archaeology
☒	☐	Animals and other organisms
☒	☐	Clinical data
☒	☐	Dual use research of concern

Methods

n/a	Involved in the study	
☒	☐	ChIP-seq
☒	☐	Flow cytometry
☒	☐	MRI-based neuroimaging

Antibodies

Antibodies used	anti-ApoAI (Abcam ab52945, Clone EP1368Y, Lot# GR3256621)<br>anti-PON1 (Abcam ab24261, Clone 17A12, Lot# GR3233596)

	anti-ApoAI (Life Technologies MIA1402, Clone 311, Lot# WL337403)<br>anti-PON1 with HRP (VWR 10408-588, polyclonal, Lot# AC032901)<br>anti-APOAI HRP (VWR 10680-872, polyclonal, Lot# 081870)
Validation	Done by the manufacturer.<br>anti-ApoAI (Abcam ab52945): https://www.abcam.com/apolipoprotein-a-i-antibody-ep1368y-ab52945.html<br>anti-PON1 (Abcam ab24261): https://www.abcam.com/pon1-antibody-17a12-ab24261.html<br>anti-ApoAI (Invitrogen MIA1402): https://www.thermofisher.com/antibody/product/ApoA1-Antibody-clone-311-Monoclonal/MIA1402<br>anti-PON1 with HRP (VWR 10408-588): https://www.biossusa.com/products/bs-5107R-HRP<br>anti-APOAI HRP (VWR 10680-872): https://us.vwr.com/store/product/16532772/anti-apoa1-goat-polyclonal-antibody-hrp-horseradish-peroxidase
