# Supplementary figures and images for: Quantifying PON1 on HDL with nanoparticle-gated electrokinetic membrane sensor for accurate cardiovascular risk assessment
Source: Nat Commun. 2023 Feb 2;14:557. doi: 10.1038/s41467-023-36258-w (PMC9895453; doi:10.1038/s41467-023-36258-w)

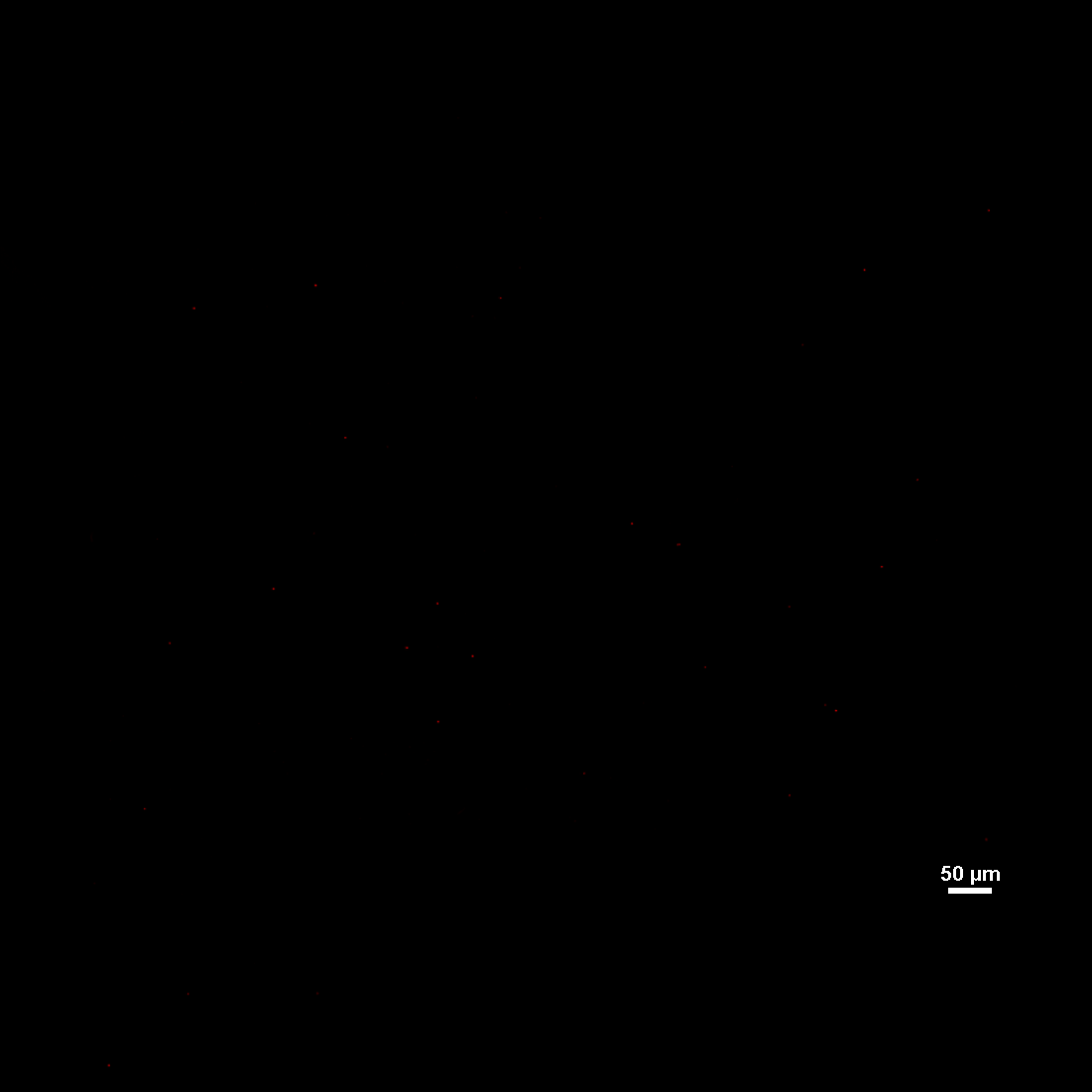

Supplement: Supplementary file 4 — Source Data [file 41467_2023_36258_MOESM4_ESM.zip › SourceData/Confocal images/Fig. 4a (PON1-PON1-HDL1pM).tif]

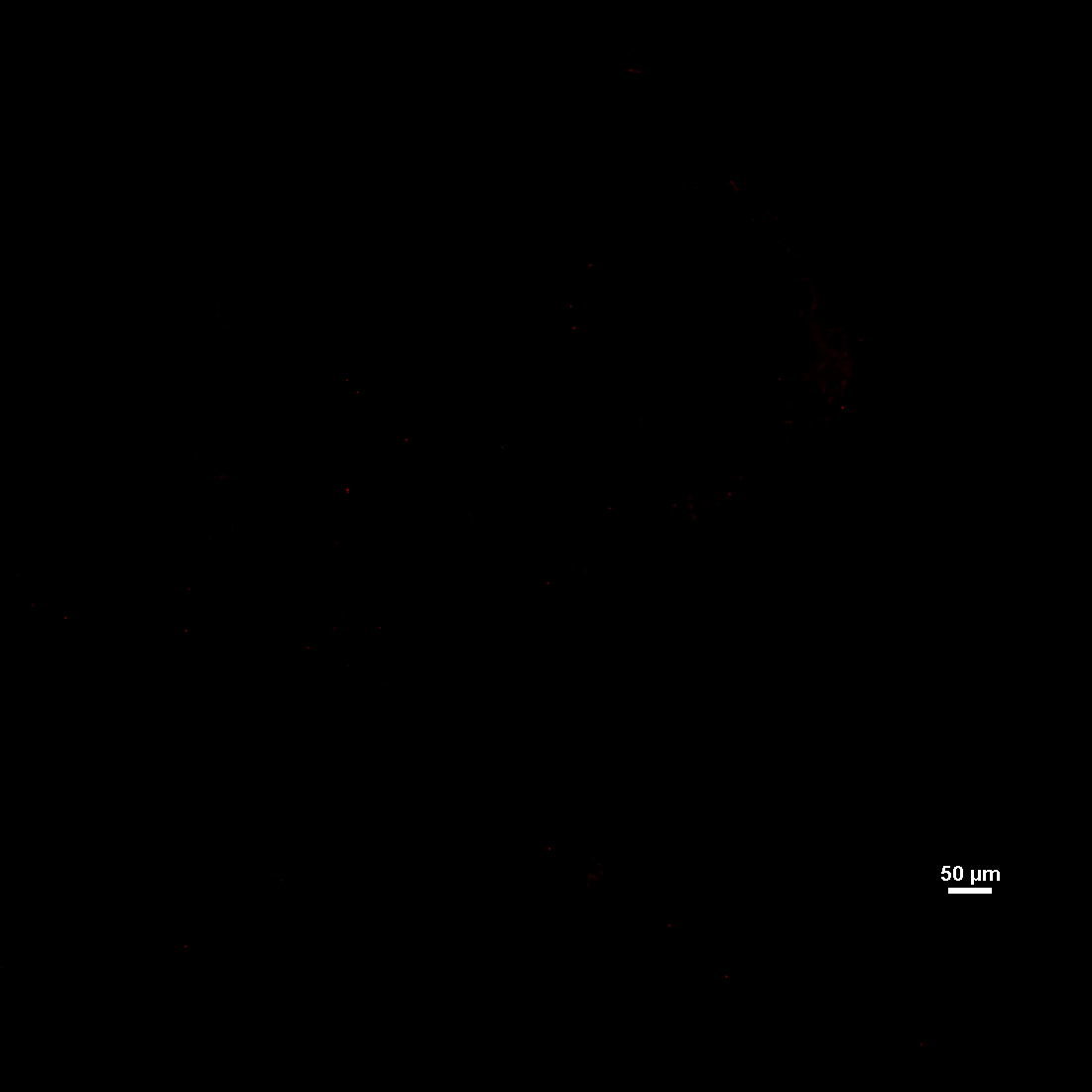

Supplement: Supplementary file 4 — Source Data [file 41467_2023_36258_MOESM4_ESM.zip › SourceData/Confocal images/Fig. 4b (PON1-HDL-pon1-10pM).tif]

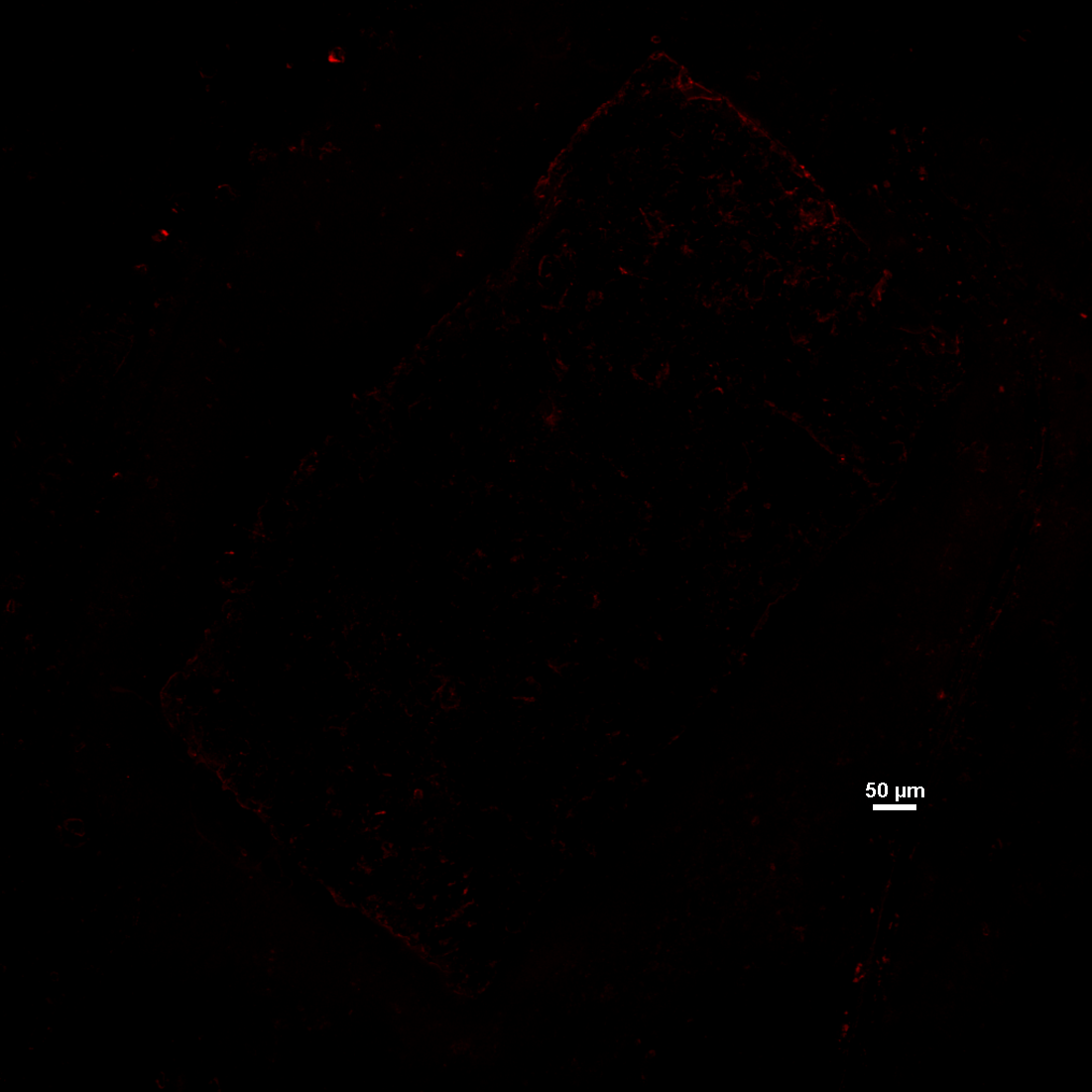

Supplement: Supplementary file 4 — Source Data [file 41467_2023_36258_MOESM4_ESM.zip › SourceData/Confocal images/Fig. 4c (PON1-PON1-0.1nM).tif]

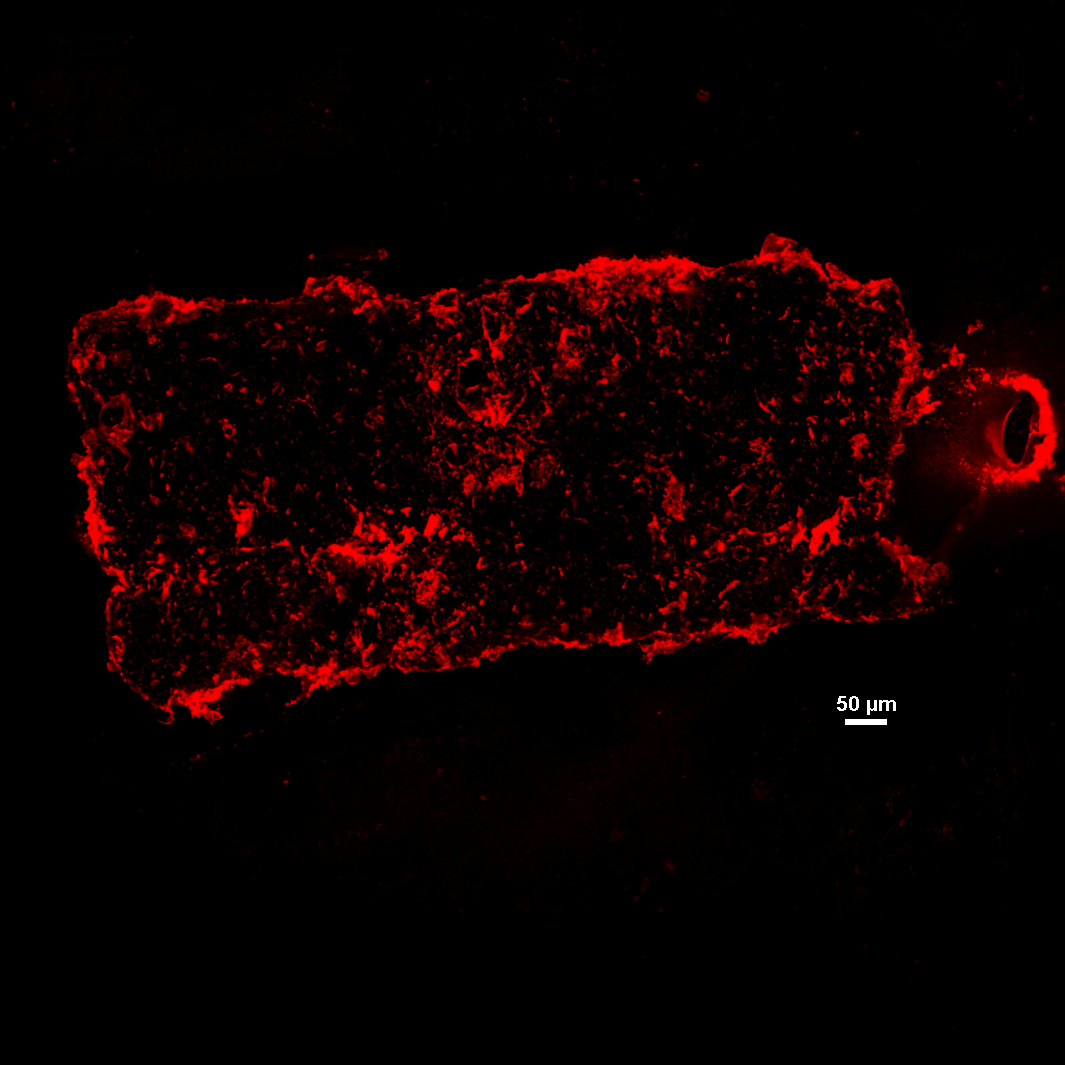

Supplement: Supplementary file 4 — Source Data [file 41467_2023_36258_MOESM4_ESM.zip › SourceData/Confocal images/Fig. 4d (PON1-PON1-1nM).tif]

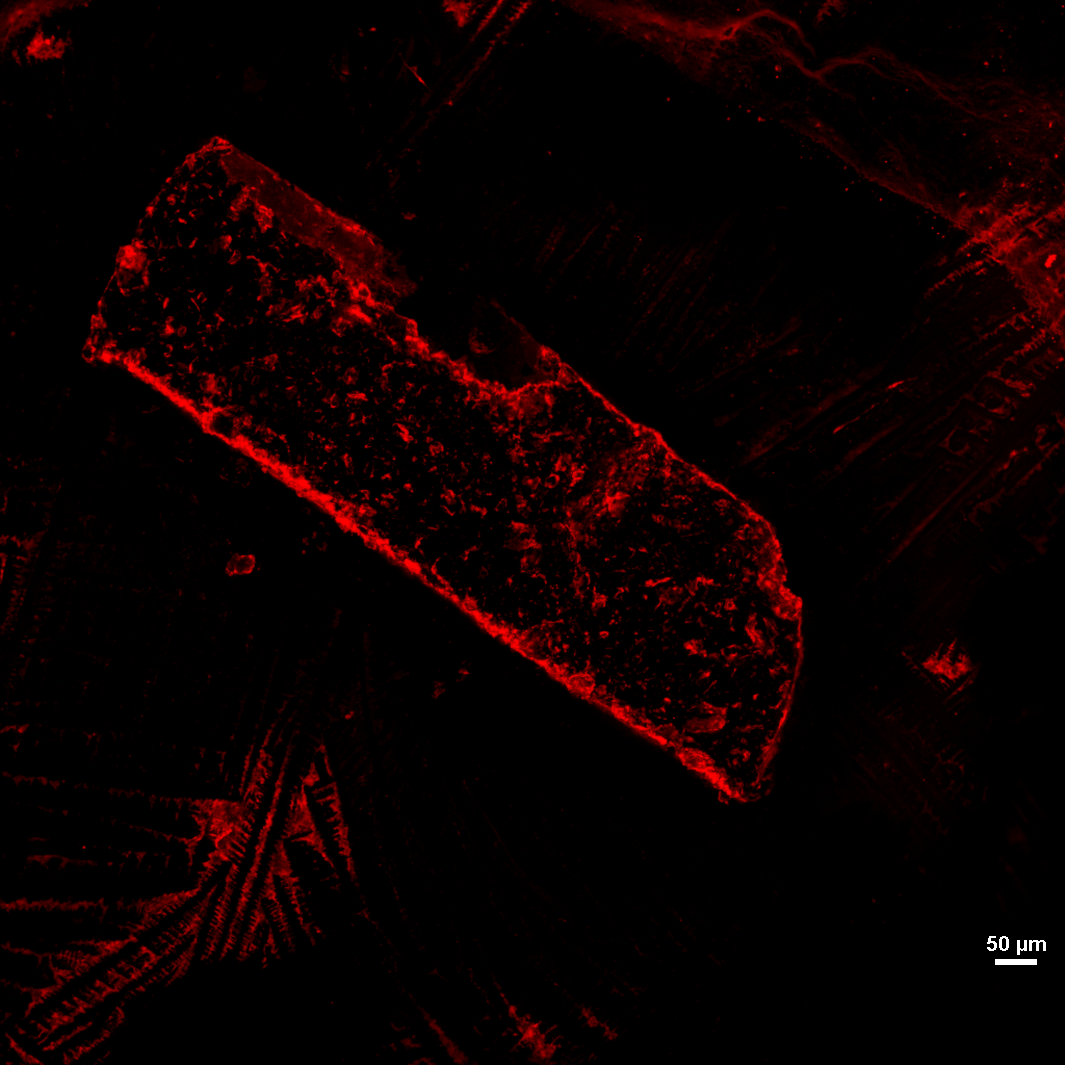

Supplement: Supplementary file 4 — Source Data [file 41467_2023_36258_MOESM4_ESM.zip › SourceData/Confocal images/Fig. 4e (PON1-PON1-10nM).tif]

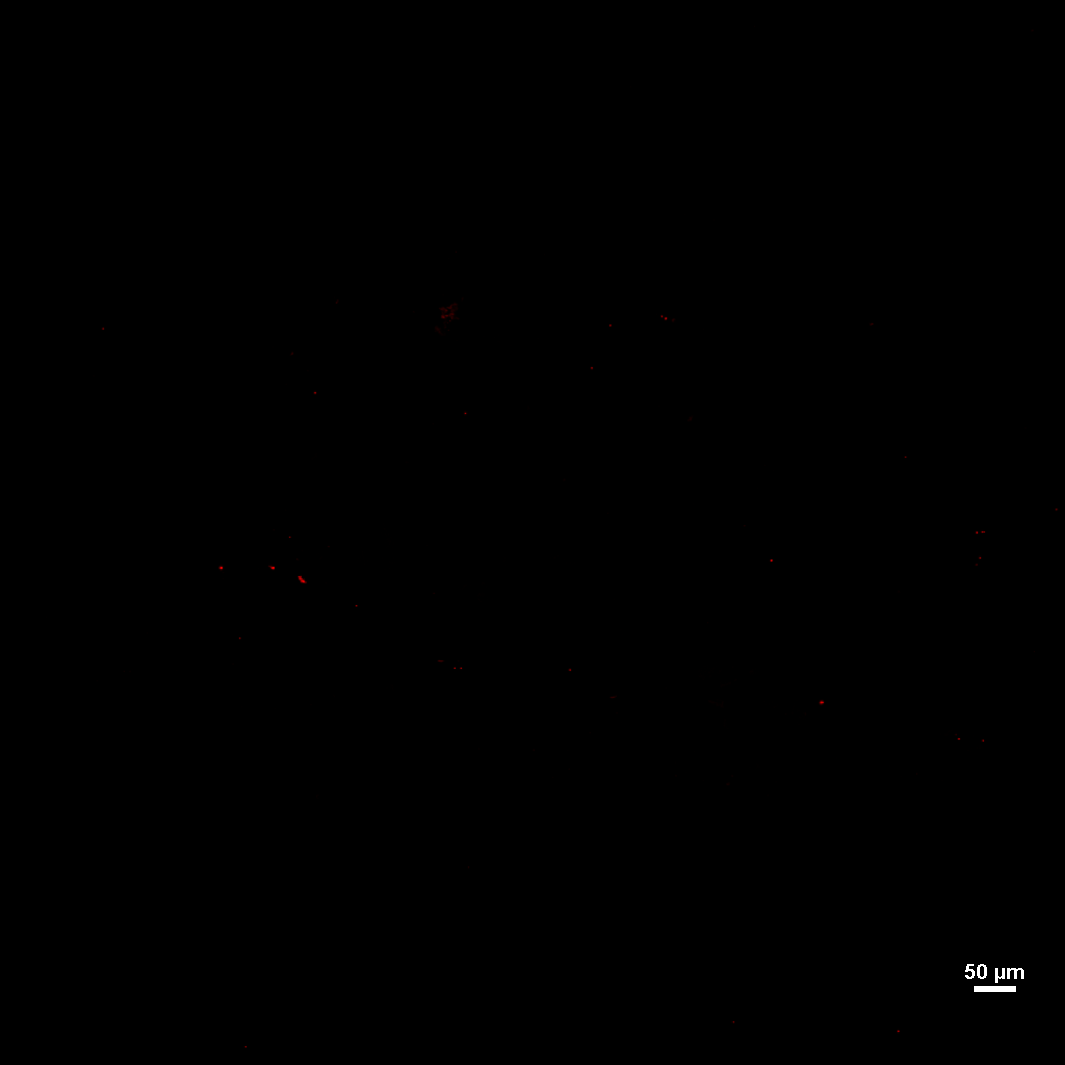

Supplement: Supplementary file 4 — Source Data [file 41467_2023_36258_MOESM4_ESM.zip › SourceData/Confocal images/Fig. 4f (cocktail-free-PON1).tif]

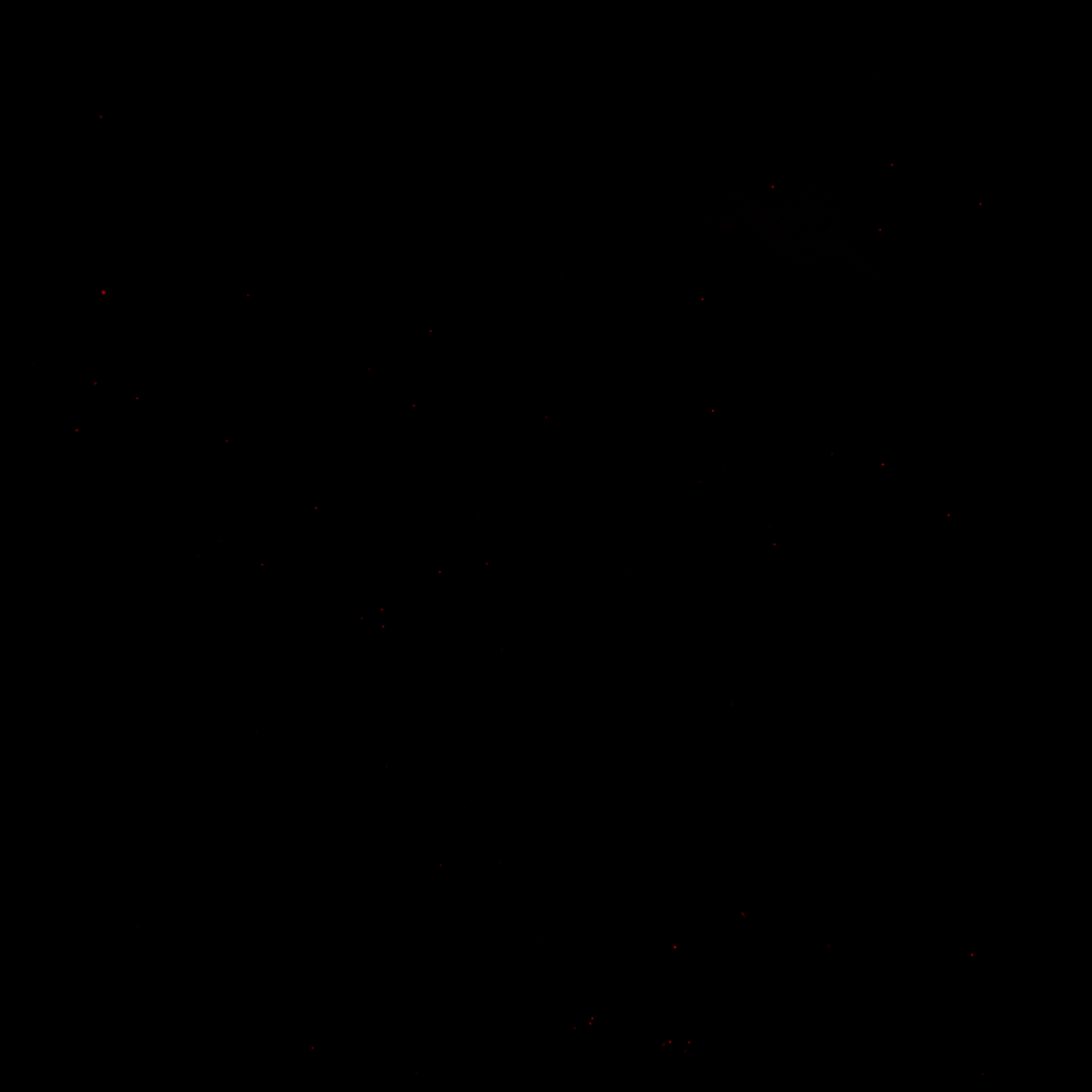

Supplement: Supplementary file 4 — Source Data [file 41467_2023_36258_MOESM4_ESM.zip › SourceData/Confocal images/Fig. 4g (wrong-captureapoB-PON1-results-0-055).tif]

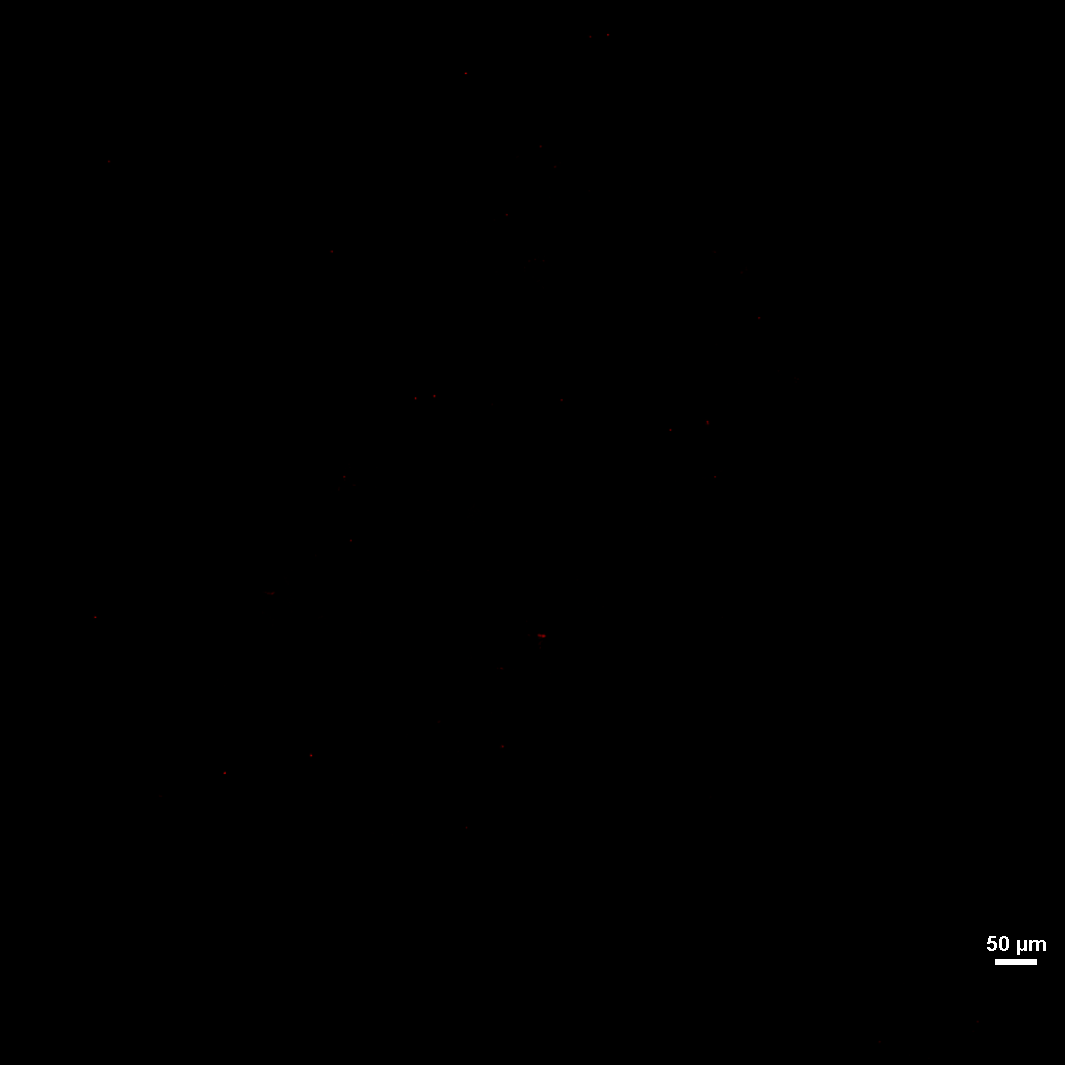

Supplement: Supplementary file 4 — Source Data [file 41467_2023_36258_MOESM4_ESM.zip › SourceData/Confocal images/Fig. 4h (ApoAI-HDL1pM).tif]

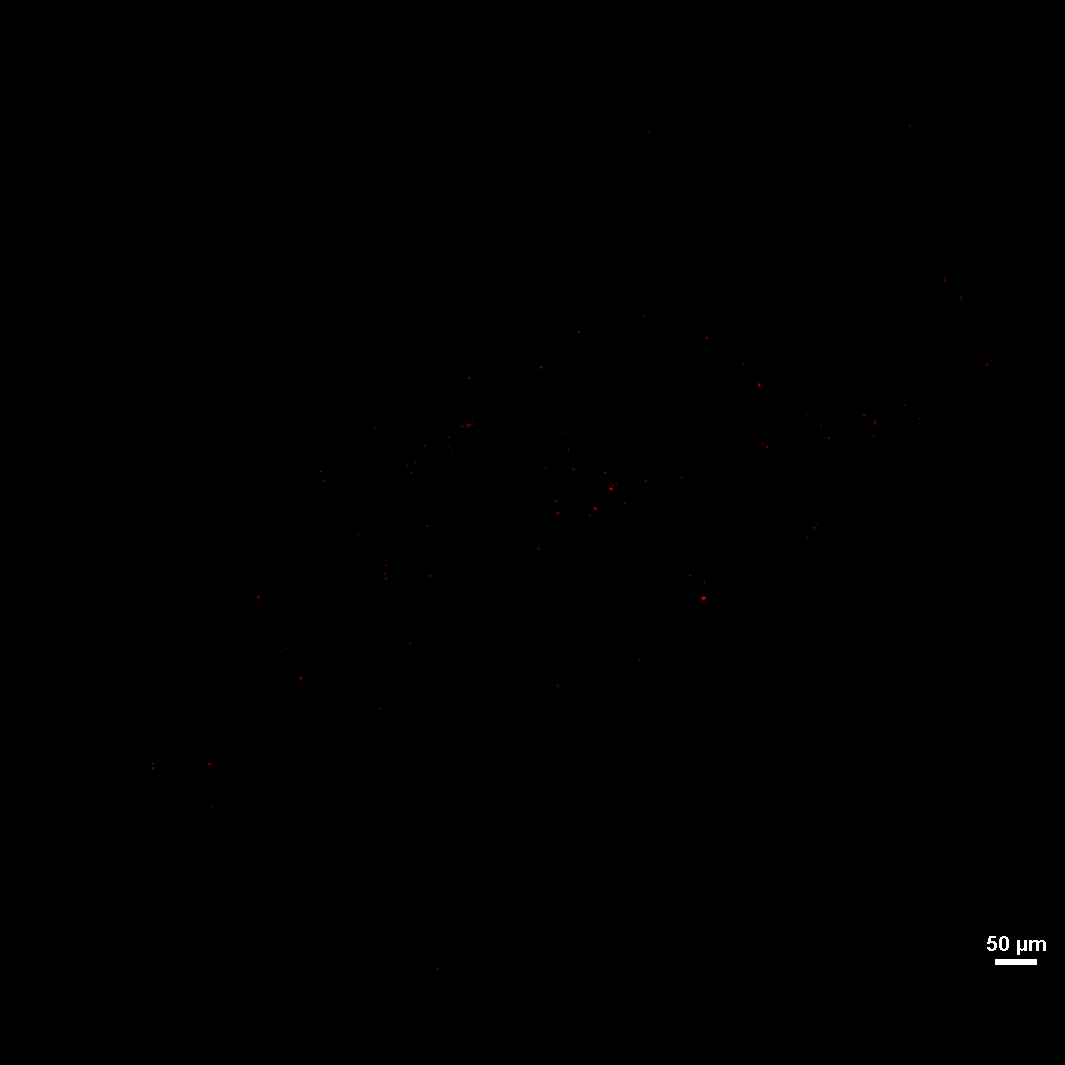

Supplement: Supplementary file 4 — Source Data [file 41467_2023_36258_MOESM4_ESM.zip › SourceData/Confocal images/Fig. 4i (ApoAI-HDL10pM).tif]

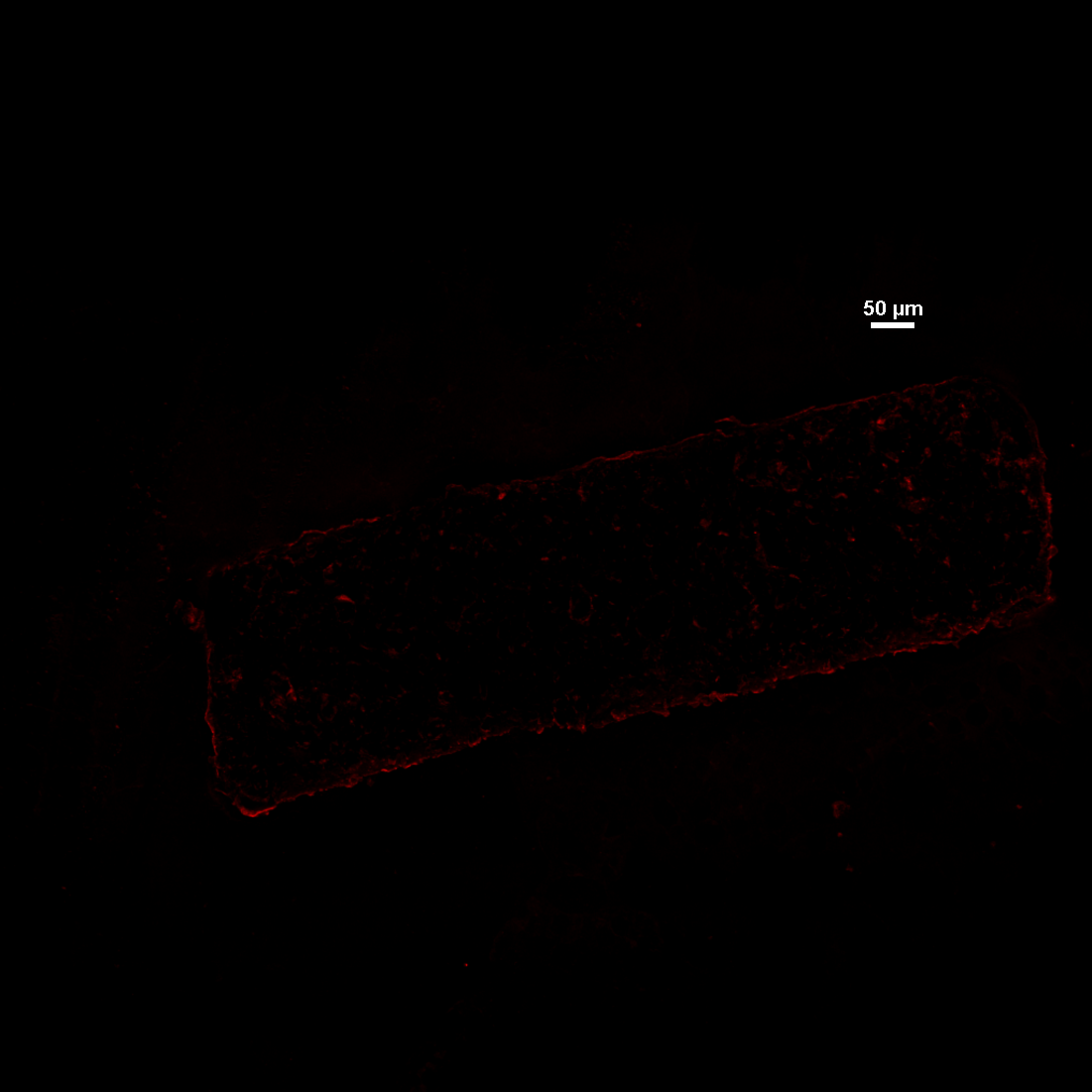

Supplement: Supplementary file 4 — Source Data [file 41467_2023_36258_MOESM4_ESM.zip › SourceData/Confocal images/Fig. 4j (apoa1-HDL100pM).tif]

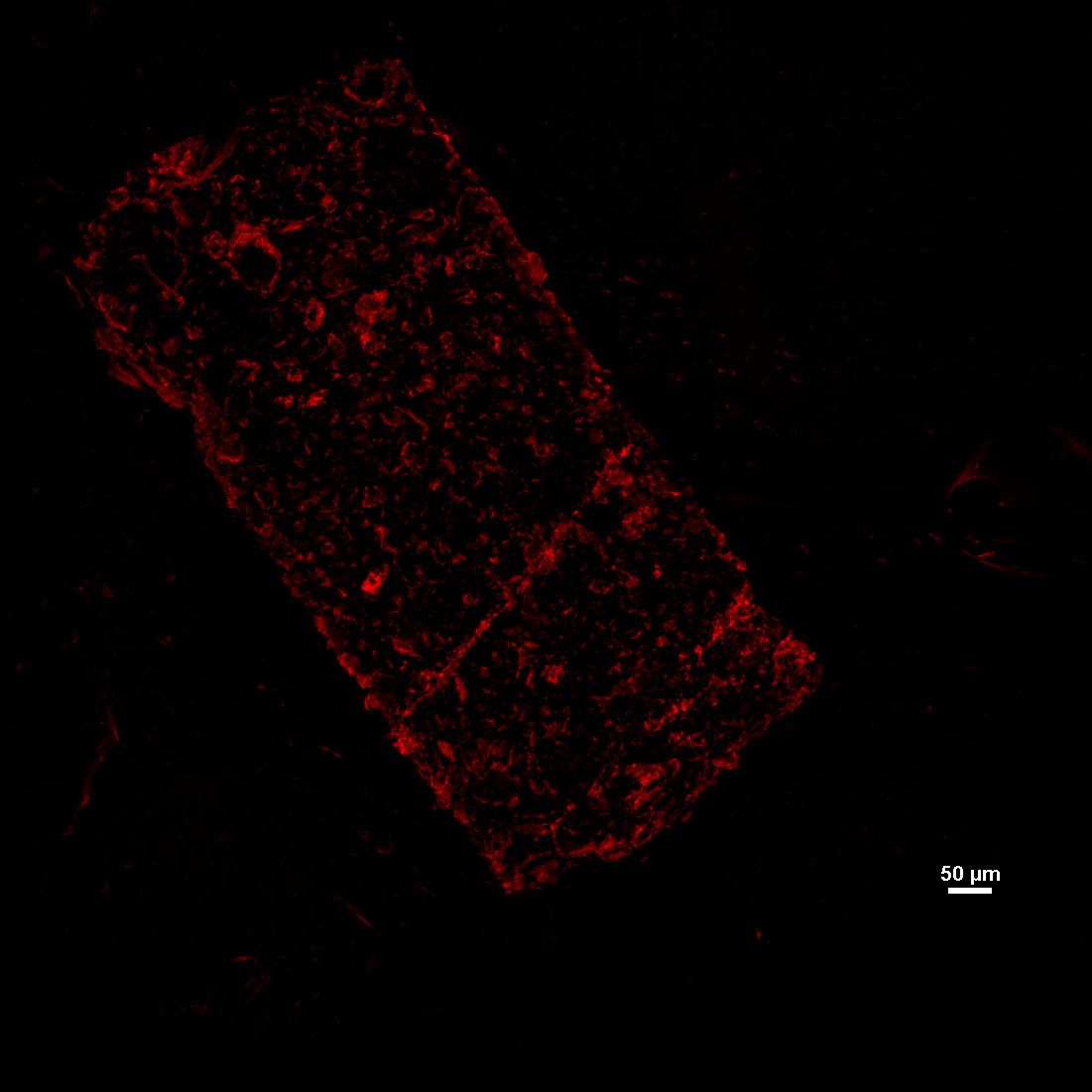

Supplement: Supplementary file 4 — Source Data [file 41467_2023_36258_MOESM4_ESM.zip › SourceData/Confocal images/Fig. 4k (apoa1-HDL1nM).tif]

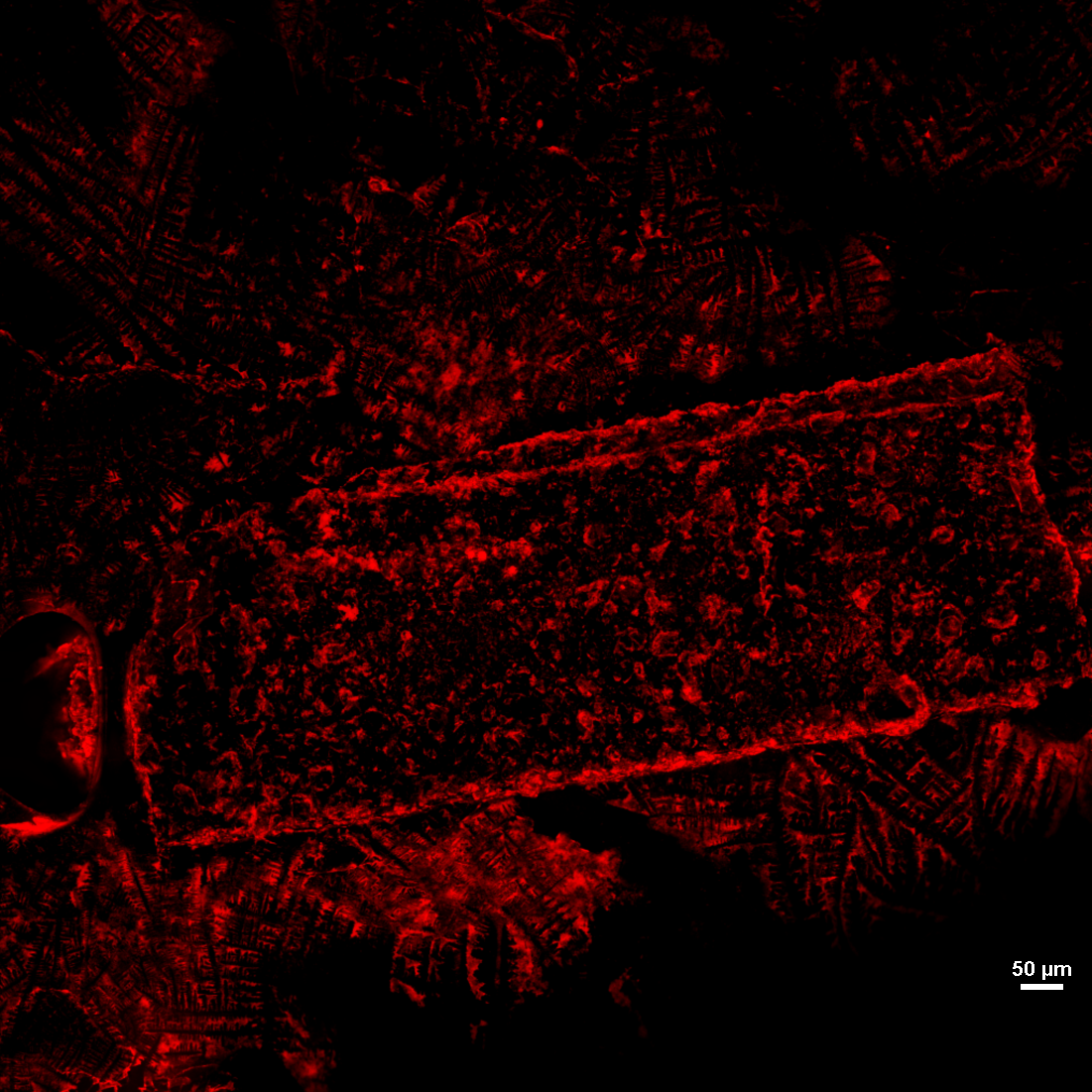

Supplement: Supplementary file 4 — Source Data [file 41467_2023_36258_MOESM4_ESM.zip › SourceData/Confocal images/Fig. 4l (apoa1-HDL10nM).tif]

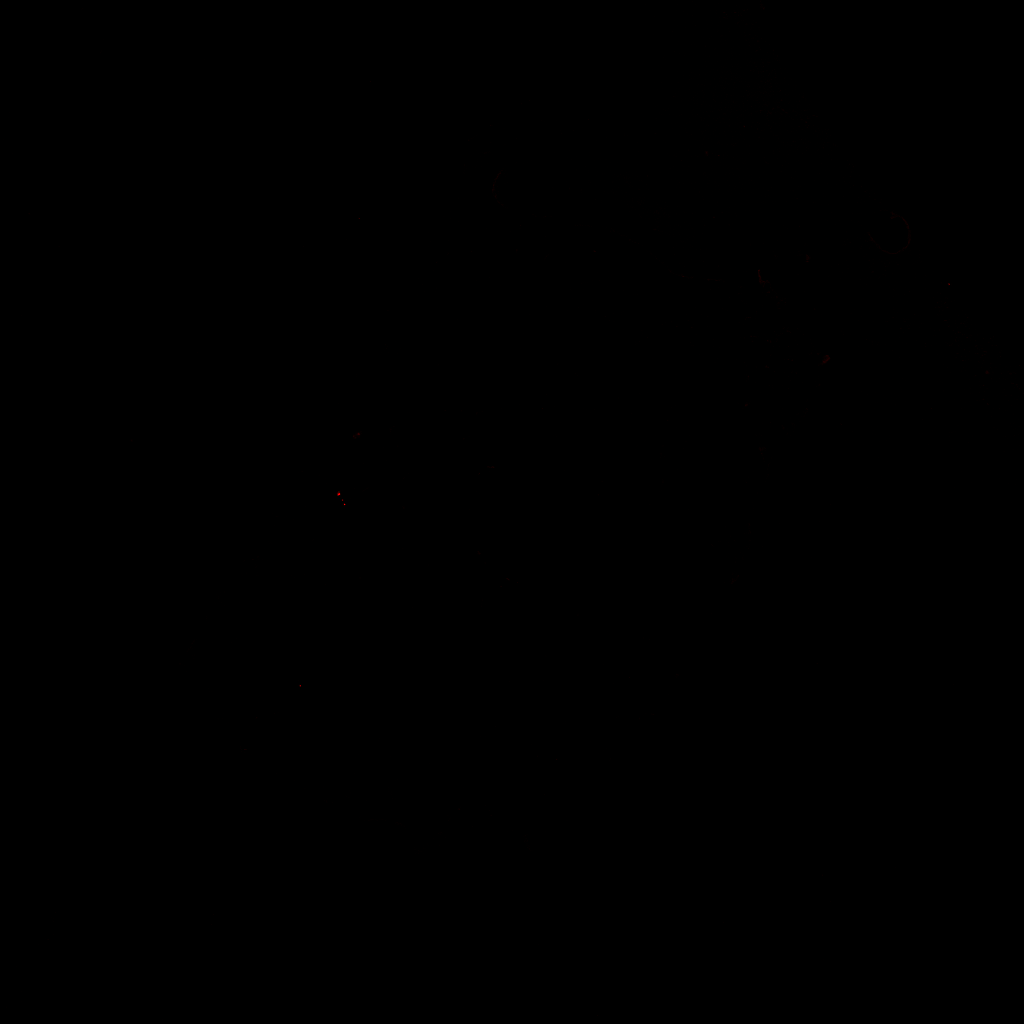

Supplement: Supplementary file 4 — Source Data [file 41467_2023_36258_MOESM4_ESM.zip › SourceData/Confocal images/Fig. 4m (cocktail-free-apoaI).tif]

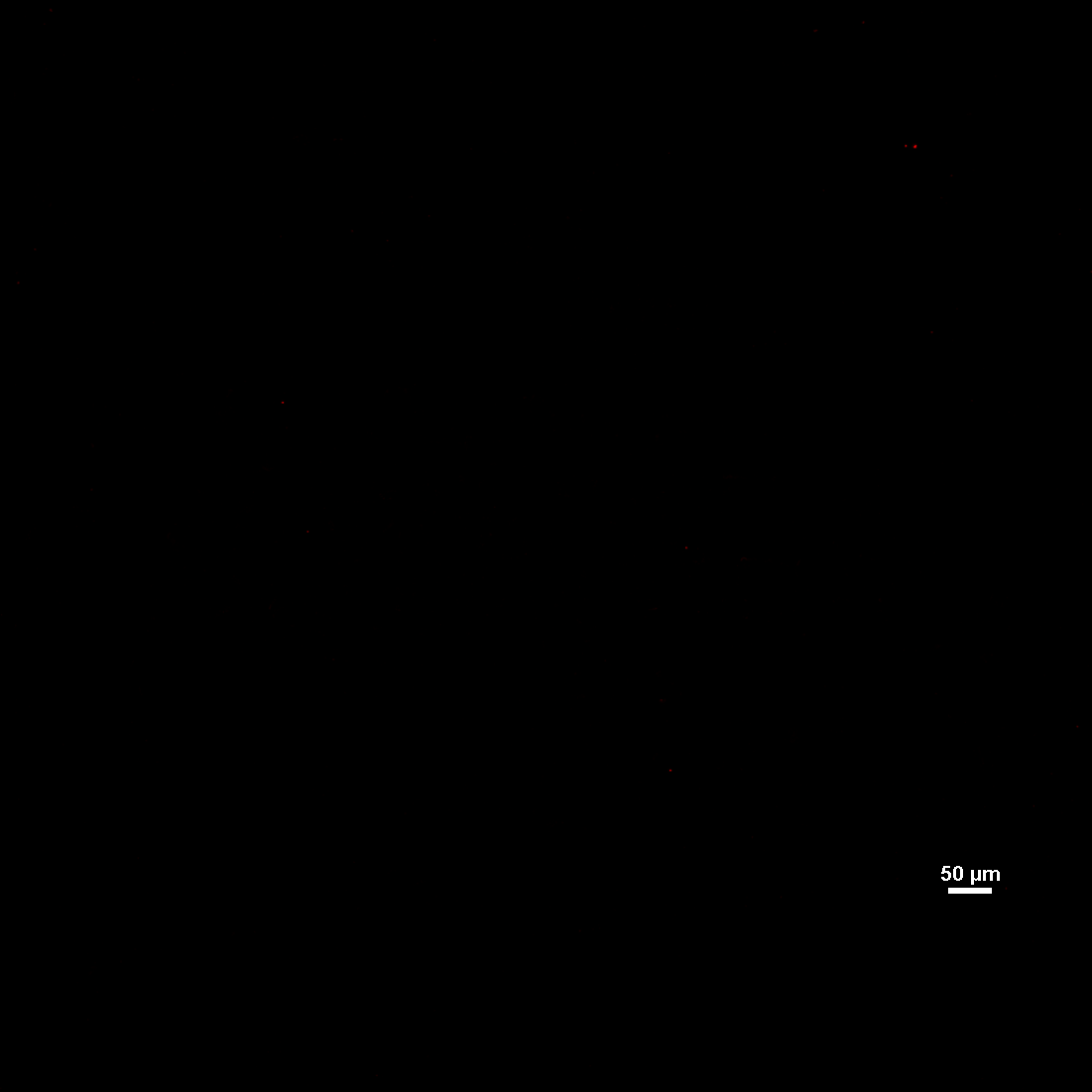

Supplement: Supplementary file 4 — Source Data [file 41467_2023_36258_MOESM4_ESM.zip › SourceData/Confocal images/Fig. 4n (wrong-captureapoB-apoAI).tif]

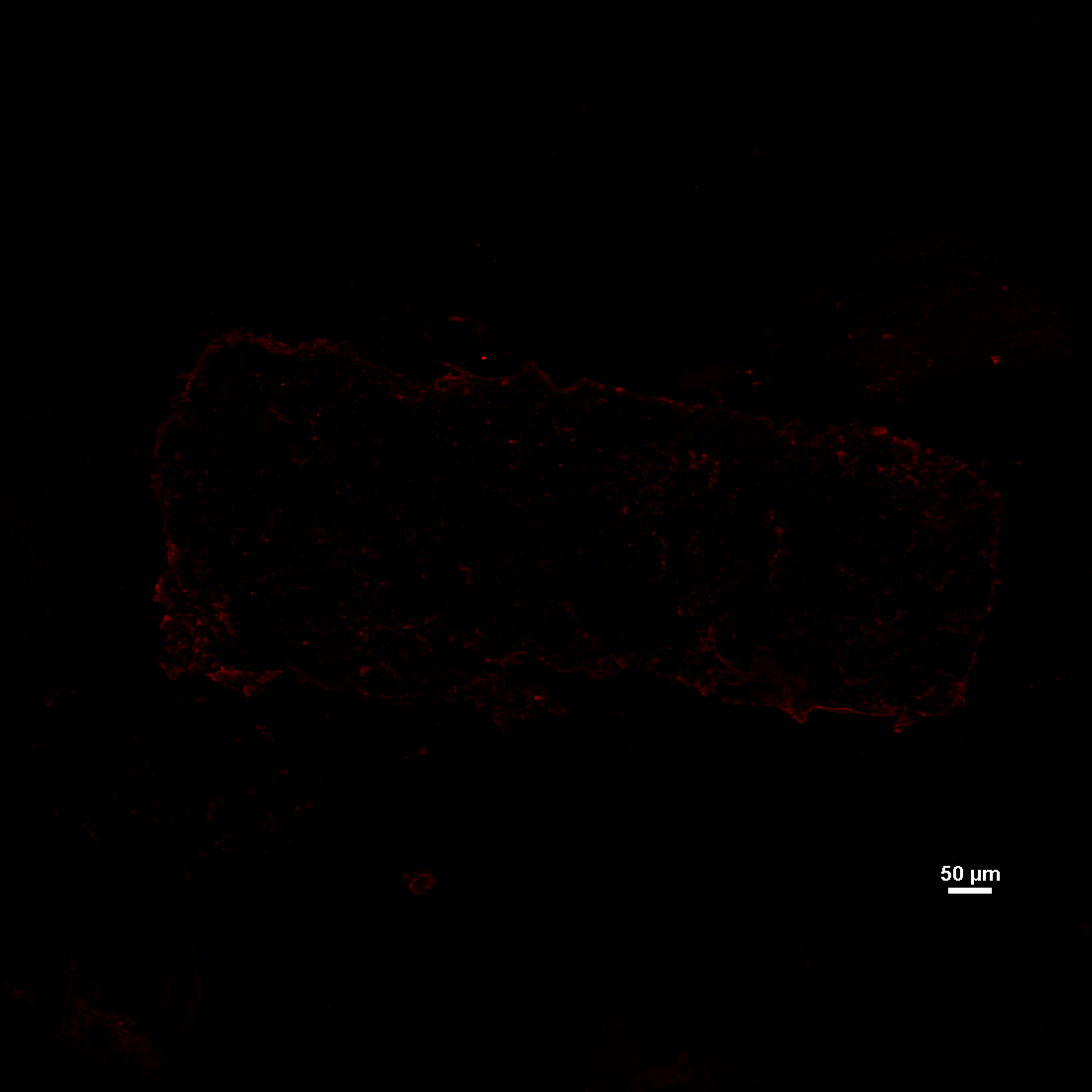

Supplement: Supplementary file 4 — Source Data [file 41467_2023_36258_MOESM4_ESM.zip › SourceData/Confocal images/Fig. 5b (apoa1-100pM-PBStreatednodetergent).tif]

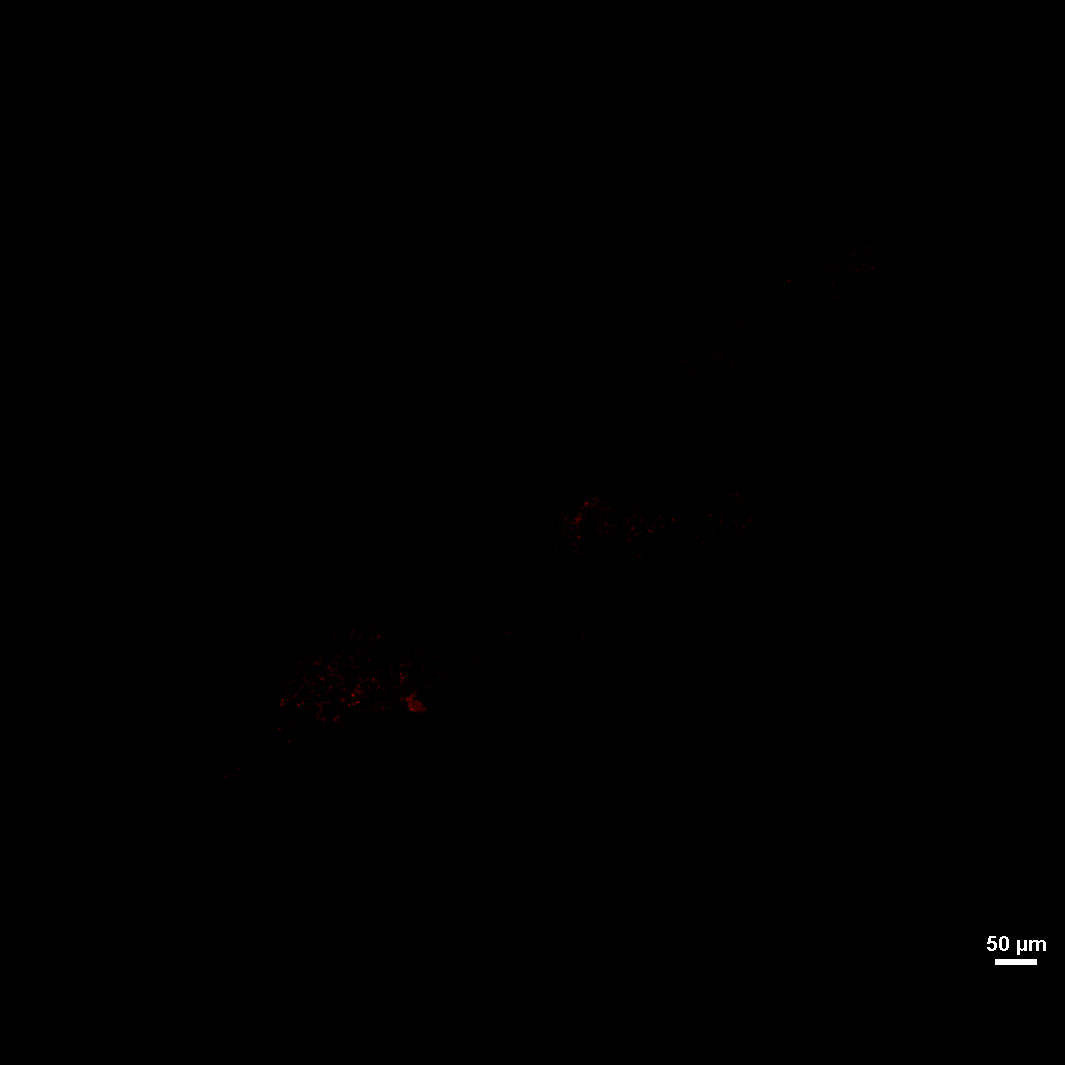

Supplement: Supplementary file 4 — Source Data [file 41467_2023_36258_MOESM4_ESM.zip › SourceData/Confocal images/Fig. 5c (on-chip-tween20-delipidation(ApoAI Si) 100pM).tif]

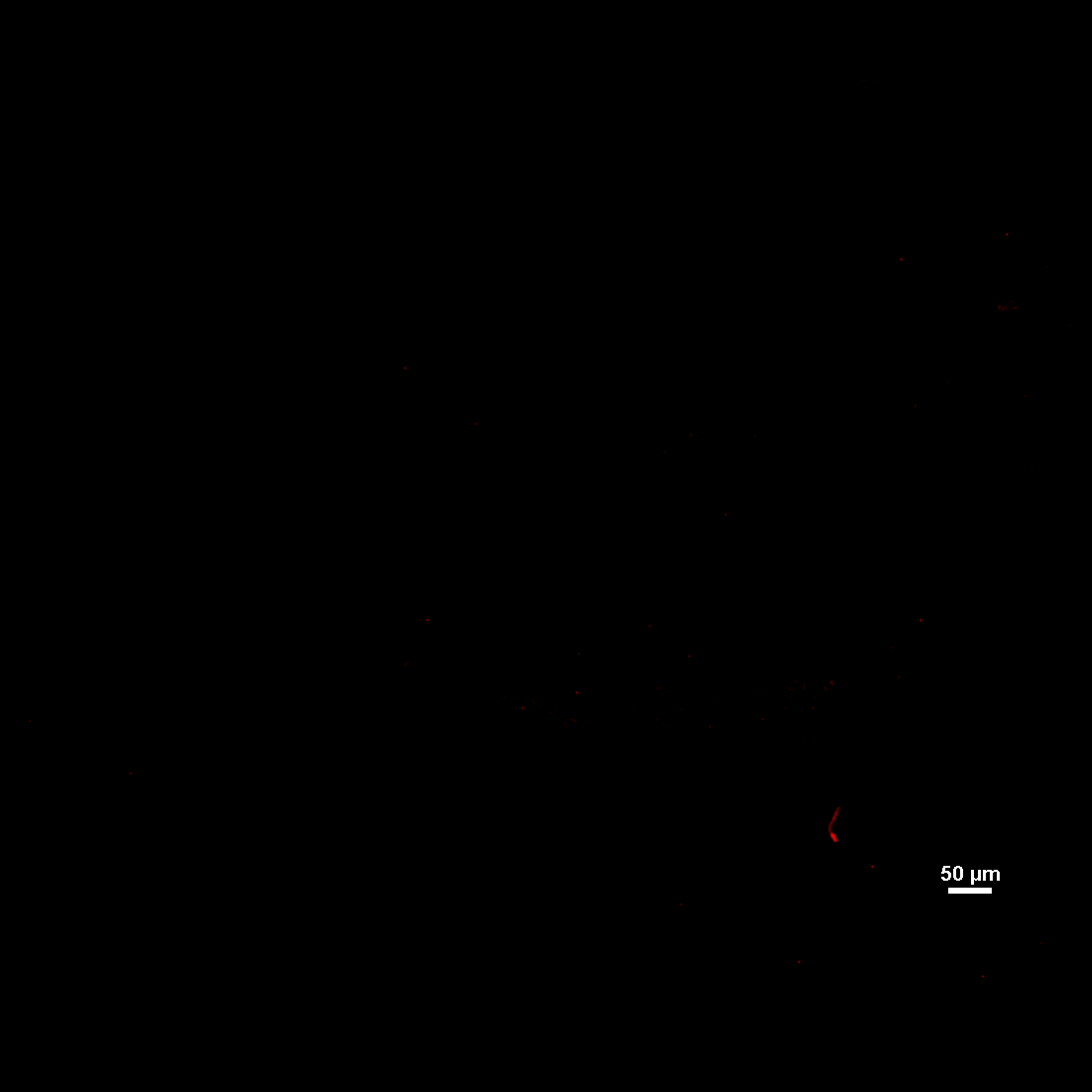

Supplement: Supplementary file 4 — Source Data [file 41467_2023_36258_MOESM4_ESM.zip › SourceData/Confocal images/Fig. 5d (PON1-delipidated-PON1HDL100pM).tif]

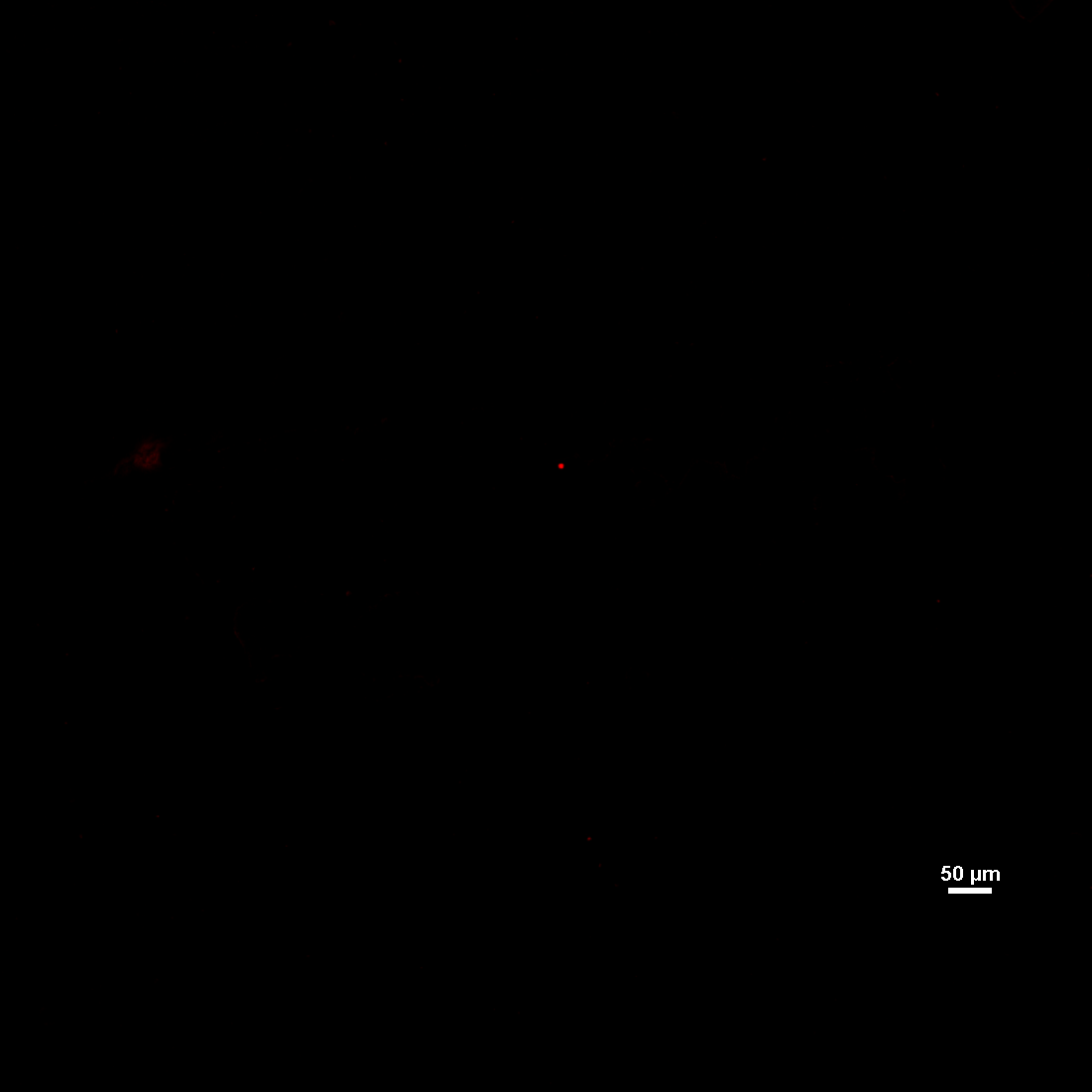

Supplement: Supplementary file 4 — Source Data [file 41467_2023_36258_MOESM4_ESM.zip › SourceData/Confocal images/Fig. 5e (ApoAI-delipidatedHDL100pM).tif]
